# Editorial: Trends in the design of functional foods for human health

**DOI:** 10.3389/fnut.2024.1393366

**Published:** 2024-03-13

**Authors:** Erick P. Gutiérrez-Grijalva, Marcela Gaytán-Martínez, Liliana Santos-Zea

**Affiliations:** ^1^Cátedras CONAHCYT-Centro de Investigación en Alimentación y Desarrollo (CIAD) A.C., Culiacán, Sinaloa, Mexico; ^2^Posgrado en Ciencia y Tecnología de los Alimentos, School of Chemistry, Universidad Autónoma de Querétaro, Santiago de Querétaro, Querétaro, Mexico; ^3^Escuela de Ingeniería y Ciencias, Tecnologico de Monterrey, Monterrey, Mexico

**Keywords:** functional foods, fermented dairy products, milk-protein fortification, phytotherapeutics, maize

There is a rising trend from consumers around the globe to acquire foodstuff that, besides nutritional value, adds beneficial health effects. People from different countries look for foods that enhance their mental performance to aid in chronic disorders such as celiac disease, metabolic syndrome, cancer prevention, and inflammation-related diseases. Moreover, ever since the COVID-19 pandemic, people have also looked for products that enhance their immune systems. On this subject, research has focused on the many aspects of the development of a functional product that includes: identifying the relationship between the food component and the health benefit, demonstration of the efficacy and intake level required for the desired effect, demonstration of its safety use, development of a suitable food vehicle for the bioactive constituent, scientific evidence for the proposed effect, and communication of the benefits to the consumers, and a follow-up to confirm the efficacy and safety of product by the consumers.

This Research Topic aimed to collect research work related to the development and design of functional foods for human health improvement. This included the evaluation of novelty ingredients, the design of foods and ingredients for a balanced microbiome, the characterization of autochthonous foods, and novelty food products designed for specific chronic health conditions. In this Research Topic, there are four manuscripts that cover the characterization of novelty food ingredients [Acosta-Estrada et al. (a); Acosta-Estrada et al. (b)], the design of new functional ingredients (Gomaa et al.), and a comprehensive literature review regarding the potential health benefits of Manilkara zapota (Rivas-Gastelum et al.).

Protein-enriched yogurt is currently being evaluated due to the increasing market of people who exercise and demand high-protein products. In this sense, the study by Gomaa et al. explored the potential of nano casein-pectin complexes to fortify yogurt. These interactions also “increase thermal stability.” However, the incorporation of these mixtures can alter the structural and sensory properties of low-fat dairy products like yogurt. Gomaa et al. prepared various NCP complex formulations and tested their physicochemical properties. The study found that fortifying skimmed milk and low-fat yogurt with the NCP complex significantly increased their nutritional value and amino acid content, while also improving their physicochemical characteristics, viscosity, and heat stability. Yogurt fortified with the NCP complex exhibited increased protein content and viscosity, with reduced syneresis, and improved lactic acid bacteria (LAB) viability, resulting in better sensory properties. Overall, the use of nanoparticles in the casein-pectin complex demonstrated potential for developing functional foods with improved nutritional value and product properties.

Acosta-Estrada et al. (a) investigated the nutritional profiles of maize tortillas, comparing those made from different genotypes, including hybrids, varieties, landraces, and dry masa flour (DMF). Moisture content is consistent across types, but significant variations exist in other nutrients, likely influenced by maize type and processing methods. High-performance hybrids and varieties (HPHV) and landraces showed the highest antioxidant capacity. DMF tortillas exhibit significantly higher levels of B vitamins, folic acid, sodium, iron, and zinc compared to HPHV. While landraces have the highest protein content, DMF tortillas have superior protein quality according to the protein digestibility-corrected amino acid score (PDCAAS). The findings provide valuable insights for producing nutritionally enhanced tortillas and related products, especially for populations with high consumption, contributing to better human health.

Acosta-Estrada et al. (b) investigates the profiles of tortillas, comparing traditional methods with those made from dry masa flour, highlighting significant variability influenced by factors like maize type and processing methods. Twenty-two samples, including hybrids, hybrid mixtures, varieties, landraces, and dry masa flours, were processed and evaluated for 70 characteristics related to maize physicochemical properties, processability, masa characteristics, and tortilla quality. Variability was observed among genotypes, particularly within landraces. High-yield hybrids and varieties demonstrated better performance throughout processing stages, while 40% of landraces yielded masa with poor machinability. Landraces exhibited higher protein content but produced tortillas with lower extensibility compared to hybrids and varieties. The research provides valuable insights into selecting suitable maize genotypes for optimal tortilla production.

Finally, the review article by Rivas-Gastelum et al. summarized recent literature Manilkara zapota, also known as “chicozapote,” is a native evergreen tree found in Southern Mexico, Belize, and Guatemala, now widely cultivated in Mexico and Southeast Asia. Traditionally, different parts of the plant have been used for medicinal purposes, with seeds aiding digestion and the bark possessing various medicinal properties. Fruits and leaves have been employed to treat a range of conditions. Chicozapote fruits are nutrient-rich and contain bioactive compounds with diverse biological activities, offering potential benefits against chronic diseases. Despite its valuable properties, the use of chicozapote is currently limited to its fresh form, resulting in waste from non-edible structures. The suggestion is to use the fruit as a raw source for creating functional foods and pharmacological products to address this issue. The article provides insights into the nutritional and phytochemical profiles of chicozapote, detailing extraction methodologies and conditions for bioactive compounds and emphasizing their broad biological effects and specific functional mechanisms.

## Author contributions

EG-G: Conceptualization, Data curation, Formal analysis, Supervision, Writing – original draft, Writing – review & editing. MG-M: Data curation, Formal analysis, Supervision, Writing – review & editing. LS-Z: Conceptualization, Data curation, Formal analysis, Investigation, Supervision, Writing – review & editing.

